# CONSORT: Different End-Points of Preoperative Nutrition and Outcome of Bowel Resection of Crohn Disease: A Randomized Clinical Trial

**DOI:** 10.1097/MD.0000000000001175

**Published:** 2015-07-24

**Authors:** Weiming Zhu, Zhen Guo, Lugen Zuo, Jianfeng Gong, Yi Li, Lili Gu, Lei Cao, Ning Li, Jieshou Li

**Affiliations:** From the Department of General Surgery, Jinling Hospital, Medical School of Nanjing University, Nanjing, PR China.

## Abstract

Nutritional therapy cannot only improve nutritional status but also reduce bowel inflammation in Crohn disease (CD). The benefits of preoperative nutritional therapy on outcomes of surgery for CD have been demonstrated. However, the ideal end-points of preoperative nutrition in CD remain elusive. We conducted this study to figure out whether improvement of malnutrition or reduction of inflammation is the better end-point of preoperative nutrition for CD.

This was a prospective, randomized study. All patients enrolled received preoperative nutrition with different end-points (improvement of malnutrition, IOM, or reduction of inflammation, ROI). The end-points were defined using serum albumin and body weight gain, and serum C-reactive protein (CRP), respectively. Postoperative complications, rate of fecal diversion, and postoperative recurrence of the disease were compared.

A total of 108 patients were randomized and 91 patients (44 in IOM group and 47 in ROI group) completed this study. It took 25.57 ± 11.68 days to achieve ROI and 45.29 ± 18.47 days for IOM (*P* = 0.0023). After nutritional therapy, serum CRP, CDAI, and serum albumin in both groups improved significantly. But patients in the IOM group had a higher albumin level and body weight gain compared with ROI group (*P* = 0.0026, *P* < 0.0001). When comparing postoperative complications, rate of fecal diversion, and postoperative recurrence, no significant differences were noted.

Compared with IOM, ROI as the end-point of preoperative nutrition had the same benefits on operative outcomes in CD patients undergoing resection, but could be achieved in a shorter time (NCT01540942).

## INTRODUCTION

Crohn disease (CD) is a chronic inflammatory transmural bowel disorder, which can affect any part of the gastrointestinal tract, especially the terminal ileum and the colon.^1^ Most patients with CD will require surgery at some point in their life. Despite an increased use of azathioprine and tumor necrosis factor (TNF)-α blockers deceases the rate of operation, 23.3% of patients cannot avoid major surgery like colectomies or resections 9 years after diagnosis,^2^ and almost half of them will undergo repeat operations for disease recurrences.^3^ Broadly speaking, the indications for surgery are specific complications of CD (mainly strictures or fistula), which cannot be controlled by drug treatment. A 2-stage procedure with a temporary diverting stoma is necessary for 39% to 51% of CD patients.^4,5^

Fifteen to thirty percent of CD patients undergoing surgery will develop postoperative complications, which were classified as major in 8% to 9% of patients.^6^ Increased inflammation process before surgery and impaired nutritional status are 2 risk factors of poor postoperative outcomes in CD.^7^ Patients with high C-reactive protein (CRP), a marker of inflammation, will suffer more postoperative complications. Also, impaired nutrition status is associated with not only high rates of postoperative morbidity, but also increased mortality for patients undergoing elective abdominal surgery.^8^ Unfortunately, as characters of CD, most CD patients suffer from severe inflammation and malnutrition, and the prevalence is even higher in CD patients requiring surgery. Therefore, preoperative management should be implemented to best prepare the CD patient prior to the surgery.

It has been demonstrated for decades that preoperative nutritional therapy can significantly improve the outcomes of surgery.^9^ In CD, nutrition plays a pivotal role. It is more than a supportive treatment that can correct malnutrition, at the same time, it can reduce the inflammatory response and induce remission without potential risk of surgery-related complications.^10^ Preoperative management including nutritional therapy can decrease postoperative morbidity and fecal diversion in CD.^11^ However, the ideal end-points of preoperative nutritional therapy remain elusive.

Taking into account the above aspects, we conducted a prospective, randomized study, in which adult CD patients undergoing resection accepted preoperative nutritional therapy with different end-points, improvement of malnutrition, or reduction of inflammation. We evaluated postoperative complications, rate of fecal diversion, and postoperative recurrence of the disease aimed at figuring out which is a better end-point of preoperative nutritional therapy for CD.

## MATERIALS AND METHODS

### Study Design

This was a prospective, single-center, single-blind, randomized trial during the period November 2011 to December 2013. The protocol was approved by the Institutional Ethics Committee of Jinling Hospital (Nanjing, China) and performed according to the Declaration of Helsinki Principles. Written informed consent was obtained from all patients. The study is registered at ClinicalTrials.gov (NCT01540942).

### Patients

Adult patients (age from 18 to 75) with a diagnosis of CD requiring elective resection due to intestinal strictures, fistula, or abdominal abscess were enrolled. The diagnosis of CD was endoscopically and histologically confirmed. Patients included should also meet the following criteria: patients had a CRP level of >8 mg/L, patients had a preoperative weight loss >5% over the past 6 months, and had a serum albumin of <35 g/L at the enrollment. Exclusion criteria included the following: patients who presented with short bowel syndrome, history of malignancy, and women who were pregnant or lactating at the time of enrollment. All patients had not been given nutritional treatment within the preceding 1 month. Patients enrolled were assigned randomly using a computer-generated sequence (www.randomizer.org) in a 1:1 ratio to an improvement of malnutrition end-point group (IOM group) or a reduction of inflammation end-point group (ROI group). Patients were blind to the end-point of nutritional therapy.

### Preoperative Management

Patients with incomplete intestinal obstruction, fistula, or abscess received exclusive enteral nutrition (EEN) using a polymeric formula (Nutricia, Amsterdam, the Netherlands). During this period, any other food and drink except water were forbidden. The enteral nutritional suspension was infused continuously through a nasogastric tube. For those who could not achieve the goal calorie intake by enteral nutrition (EN) alone, EN combined with parenteral nutrition (PN) was implemented. Patients with complete intestinal occlusion or complete food intolerance were given a total parenteral nutrition (TPN). The ingredients of PN were decided by the dietician and mixed by nurses in a sterilizing room. The daily calorie intake was 20 to 25 kcal/kg body weight. Patients were withdrawn if treatment failed.

Sulfasalazine or mesalazine, azathioprine, and anti-TNF-α therapy were stopped after the patient was hospitalized and weaning of steroids was achieved before surgery. Smoking was also ceased as soon as the patient was hospitalized. Depending on the location and size/type of the abscess/fistula, a continuous irrigation drainage was performed on patients presented with abscess/fistula.

### Surgical Procedure

All operations were carried out by the same surgeon team with a high level of expertise in CD using open surgical resection. The resections were performed with a security margin of 2 to 3 cm. Stapled side-to-side anastomosis using linear cutter 50 mm and linear stapler 75 mm was applied to re-establish the intestinal continuity. The indication for a temporary diverting stoma was: presence of abscess irrespective of drainage before surgery, and/or severe intestinal edema, and/or ≥2 anastomosis performed. Delayed anastomosis was performed 3 to 6 months later.

### Postoperative Management

EEN was given 2 to 3 days after surgery and used for at least 1 month, and then patients returned to a normal diet in a 1-week schedule. All patients received azathioprine tablets 2.0 to 2.5 mg/kg/day orally as the maintenance therapy, which was started within 2 weeks after operations.

### Study Assessments

All patients’ baseline characteristics were recorded. Serum albumin, CRP level, body weight, and Crohn Disease Activity Index (CDAI) score were measured at the enrollment and also the day prior to the surgery. During the preoperative period, blood samples were collected and tested every 2 to 3 days. Body weight was monitored daily. After surgery, patients were followed in-hospital daily until discharge and then study visits took place at months 1 (after surgery), 2, 4, 6, 8, 10, and the final 12. The CDAI score and CRP were recorded at each visit point. At 12 months, endoscopy was performed. The endoscopist was blind to treatment assignment. Patients requiring operations or modification of drugs due to recurrence were withdrawn.

## DEFINITIONS

### Preoperative Nutrition Treatment Failure

End-point of therapy could not be achieved over 3 months.

### End-Point of Preoperative Nutritional Therapy:

Improvement of malnutrition: serum albumin >35 g/L and the increase of body weight >3 kg;

Reduction of inflammation: CRP <8 mg/L.

### Primary Outcome

Postoperative complications: postoperative complications were defined as any complication within 4 weeks after operation.

Rate of temporary diverting stoma: intentionally performed at the time of surgery and not for anastomotic leakage in postoperative period.

### Secondary Outcome

Clinical recurrence: clinical recurrence was defined as a CDAI score >150.

Endoscopic recurrence: endoscopic recurrence was defined as Rutgeerts score ≥i2. Rutgeerts score was as follows: i0, no lesions; i1, ≤5, aphthous lesions; i2, >5, aphthous lesions with normal mucosa between the lesions or skip areas of larger lesions or lesions confined to the ileocolonic anastomosis (ie, <1 cm in length); i3, diffuse aphthous ileitis with diffusely inflamed mucosa; i4, diffuse inflammation with larger ulcers, nodules and/or narrowing.^12^

### Sample Size Calculation

Sample size calculation was based on our data of historical comparison. The rate of postoperative complications was 11.2% in ROI group and 36.7% in IOM group. Forty-three patients in each group were needed to detect a difference in postoperative complications with a 80% power and a 2-sided 5% significance level.

### Data Analysis

Continuous variables were expressed as mean ± standard deviation (SD), and categorical data were expressed as frequency. Unpaired/paired *t* tests or Chi-square test were performed for 2 groups’ continuous or categorical data comparison (SPSS 17.0, Chicago, IL and GraphPad Prism 5, San Diego, CA). *P* values <0.05 were considered significant.

## RESULTS

### Baseline Characteristics

In all, 108 patients were eligible and randomly assigned to 2 groups. Ten and 7 patients withdrew due to treatment failure in IOM and ROI group, respectively. Finally, 91 patients were included in the analysis of surgical outcomes: 44 in IOM group and 47 in ROI group. During the postoperative follow-up period, 2 patients in IOM group and 3 in ROI group withdrew before 1 year due to recurrence of CD. One patient switched to infliximab therapy, and another one received operation in IOM group, whereas 2 patients switched to infliximab therapy, and 1 patient received operation in ROI group. All these 91 patients underwent endoscopy evaluation and were included in the analysis.

The baseline characteristics of 2 groups are shown in Table 1. There was no difference in age, sex, body mass index, smoking, duration of CD, disease location, disease behavior, indications for resection, serum CRP, albumin, and CDAI score in the 2 groups.

**TABLE 1:**
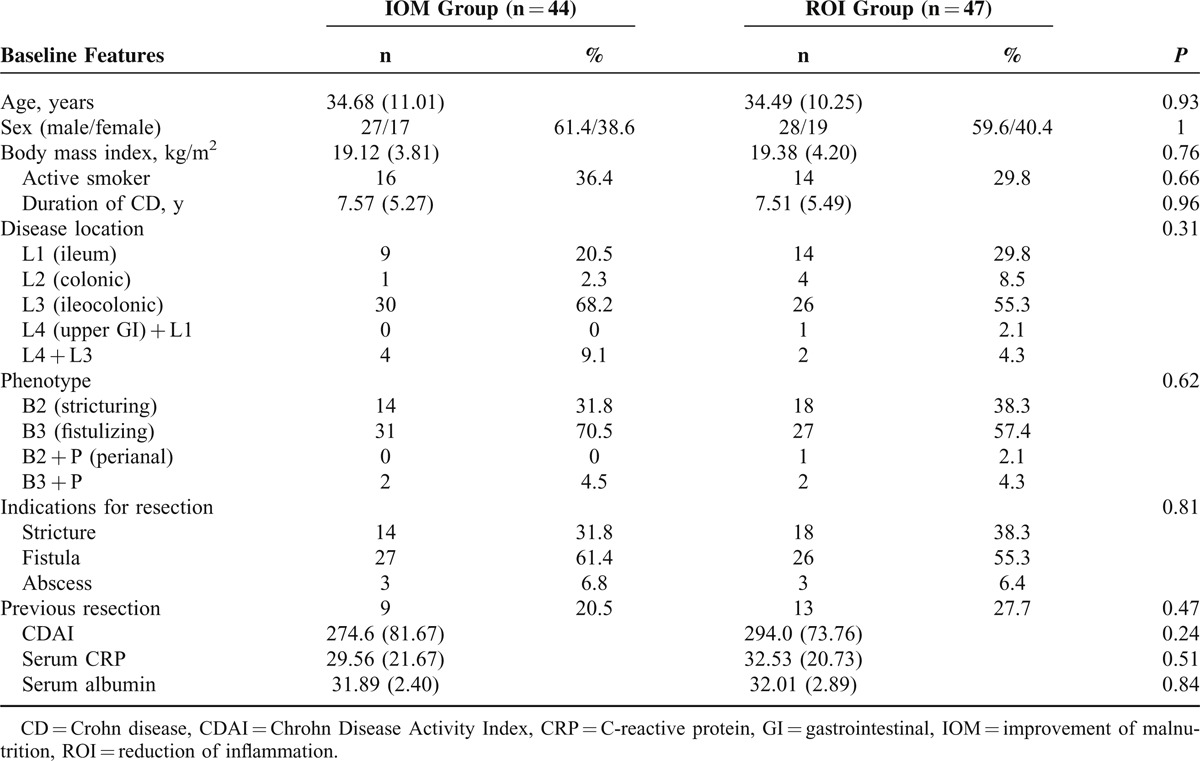
Patient Baseline Characteristics

### Preoperative Management

Preoperative managements are reported in Table 2. No difference was noted between these 2 groups. Most patients (86.4% in IOM and 85.7% in ROI group) received EEN, whereas 2 patients in the IOM group and 3 patients in ROI group were treated with TPN due to complete intestinal occlusion. The approach of nutritional therapy, drainage of abscess/fistula, and cessation of drugs were all similar in the 2 groups.

**TABLE 2:**
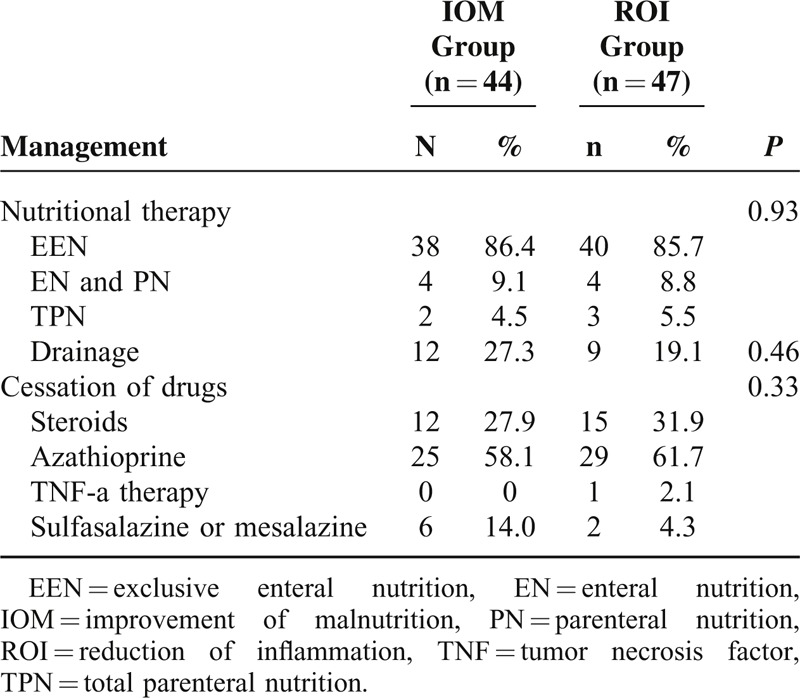
Preoperative management

### End-point of Preoperative Nutritional Therapy

**FIGURE 1:**
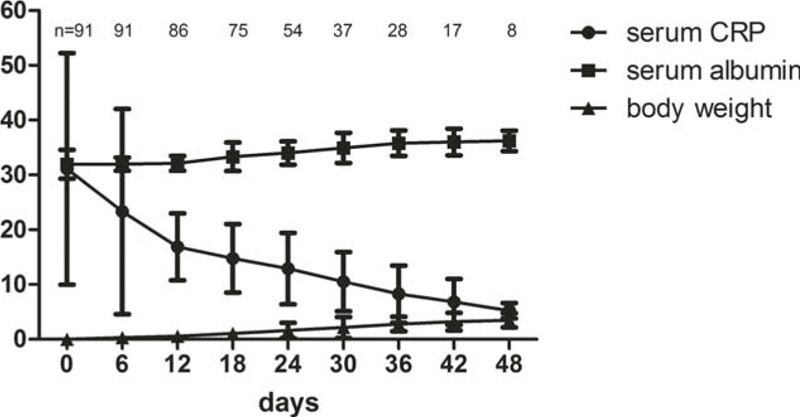
Changes of serum albumin levels (gram per liter), serum C-reactive protein levels (milligram per liter) and body weight during preoperative nutrition therapy. n =  number of patients who did not finish the preoperative treatment at each time point. Data were expressed as mean ± standard deviation.

As shown in Figure 1, following instigation of nutritional therapy, mean serum albumin (gram per liter) rose in parallel with the fell of mean serum CRP (milligram per liter). The body weight (kilogram) also increased stably. It took 25.57 ± 11.68 days to achieve the end-point of ROI and the time was 45.29 ± 18.47 days for the end-point of IOM (*P* = 0.0023). In ROI group, 12 (25.5%) patients also met the criteria of IOM, whereas in IOM group, 41 patients (93.2%) had a normal serum CRP level at the day of surgery. When the end-points were achieved, serum CRP, CDAI, and serum albumin in both groups improved significantly. But patients in the IOM group had a higher albumin level and body weight gain (Table 3).

**TABLE 3:**
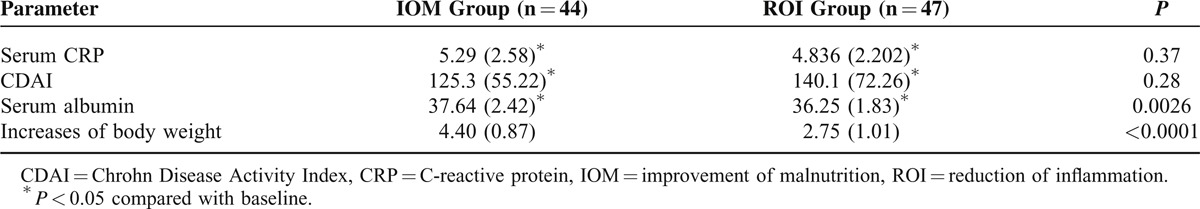
Clinical parameters after nutritional therapy

### Postoperative Complications and Temporary Diverting Stoma

Table 4 provides data on the surgical outcomes. Five patients (11.4%) in IOM group and 7 (14.9%) patients in the ROI group developed wound infections. Two patients in IOM developed anastomotic leakage and one of them needed reoperation. One patient in ROI group suffered from anastomotic leakage and another one developed intra-abdominal abscess. Both of them were treated conservatively. Fecal diversions were all temporary. No difference in surgical outcomes was noted in the 2 groups.

**TABLE 4:**
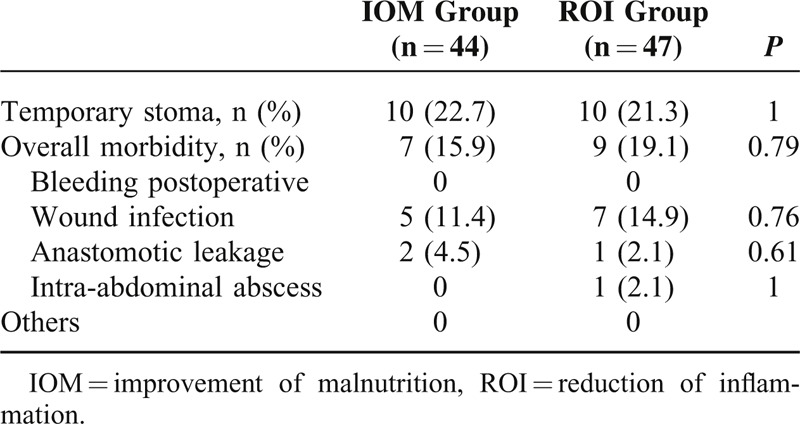
Surgical Outcomes

### Recurrence

All of the 91 patients underwent ileocolonoscopy. Five patients had ileocolonoscopies before 12 months because of early withdrawal. Seven of the 44 (15.9 %) patients in IOM group had endoscopic recurrence, compared with 6 of 47 (12.8 %) patients in the ROI group (*P* = 0.77).

Nine of 44 (20.5 %) patients in the IOM group had a clinical recurrence, whereas the clinical recurrence rate in ROI group was 12.8% (6/47) (Table 5).

**TABLE 5:**
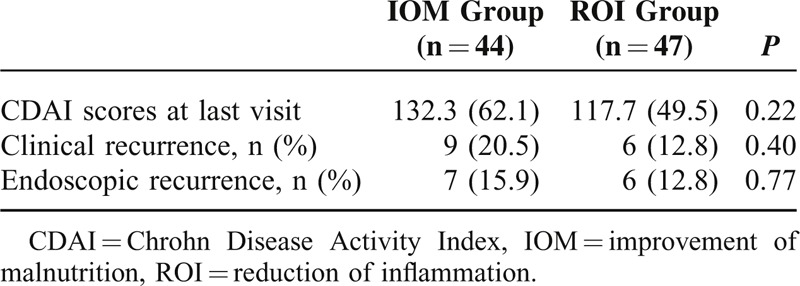
Postoperative Recurrence

## DISCUSSION

Emergency surgery is seldom necessary in CD,^13^ as a result, most patients can be best prepared for the surgery via preoperative management including preoperative nutritional therapy. To our knowledge, this is the first study to compare the influence of different preoperative nutritional therapy end-points on the surgical outcomes and recurrence of disease in CD. Our results suggested that with the same preoperative management, it needed much more time to achieve the end-point of IOM. Although patients in IOM group could get a higher serum albumin level and more gain of body weight, no difference in postoperative complications and disease recurrence was observed compared with patients in ROI group.

Malnutrition is a significant problem in CD patients. Twenty percent to 80% patients with inflammatory bowel disease will develop malnutrition.^14,15^ Serum albumin and body weight are 2 common tools used to assess nutritional status in patients. The prevalence of hypoalbuminemia is 25% to 80% in hospitalized CD patients.^16^ Low serum albumin level has been demonstrated to have a major impact on postoperative complications in cancers.^17,18^ Yamamoto et al^19^ also found that preoperative low albumin level increased the risk of septic complications after surgery in CD. However, recent studies demonstrate that the rate of albumin synthesis is affected by not only nutrition but also inflammation.^20^ Increased levels of interleukin-6 and TNF-α, which are main inflammatory mediators in CD, may decrease albumin synthesis.^21,22^ This indicated it is not sufficient to use serum albumin alone as the marker of nutritional status. Generally, interval reassessment of weight gain is accepted as the primary determinant of successful nutritional support. The improvement of weight gain can be achieved through a short-term nutritional therapy and lead to an improved outcome.^23^ For this reason, in this study, we used serum albumin level and increase of body weight to assess the nutritional status.

Inflammation is another independent risk factor of the poor postoperative outcome. We found that patients with active disease at the time of surgery experienced more postoperative complications.^24^ CRP is one protein participating in the acute phase of the inflammatory response. Due to its rapid response and short half-life, CRP has become a useful marker of inflammation. Patients with CD have a higher CRP production compared with patients with other inflammatory bowel disease, and in CD, CRP level correlated well with disease activity. Besides, CRP has been proven to be a good marker for risk of surgery and treatment response in CD.^25,26^

The effect of nutritional therapy, especially EN, on inducing remission in CD has been confirmed for decades. Nearly 90% CD patients can respond to nutritional therapy. Through nutritional treatment, both CDAI and CRP can decrease to the normal range along with the improvement of serum albumin and body weight.^27^ In the present study, EN was our first choice to treat patients. The results also suggested that nutritional therapy was effective both in improving nutritional status and decreasing serum CRP levels. However, it took more time to accomplish the goal of nutritional improvement than to reduce CRP levels. Our results found that serum albumin levels rose in parallel with the fall of CRP levels. The body weight increased stably but slowly. There are different reasons for developing malnutrition in CD, and inflammation is one of them. Increased levels of TNF and other cytokines may result in anorexia, which is one prominent cause.^28^ Also, patients may have inadequate intake and extra loss of nutrients due to abdominal symptoms. In addition, persistent inflammation will increase the consumption of nutrients. This may be one reason why nutritional improvement, especially gain of body weight, lagged behind control of inflammation. The extra loss of nutrients due to fistula (58/91 in our study) may also lead to the slow increase of body weight.

Although longer duration of nutritional therapy brought more serum albumin and body weight increase, no extra improvement in postoperative complications and rate of fecal diversion were found. Regarding recurrence of disease, there was also no meaningful difference between the 2 groups. As showed in the results, in the ROI group, patients also got a significant improvement in albumin and body weight despite they did not meet the criteria of the IOM end-point. This suggested that extra increase of albumin and body weight in IOM group may not benefit the surgical outcomes. The rate of overall complications in our study was 17.6 (16/91), and 75% (12/16) of them were classified as minor (Grade I and Grade II according to Clavien-Dindo Classification of Surgical Complications).^29^ This result is superior to those reported in a meta-analysis, in which the postoperative morbidity rate was 20.2% for open surgery in CD.^30^ We believed that preoperative nutrition in this study mainly contributed to this good outcome. Compared with the baseline, after nutritional treatment, patients had a much better inflammatory and nutritional status in terms of serum CRP and albumin and body weight. Other preoperative management like weaned-off corticosteroids, azathioprine, and anti-TNF-α agents, and abscess/fistula drainage may be also linked to the low rate of postoperative morbidity. Preoperative corticosteroids increased the risk of all postoperative complications and of infectious complications,^31^ and abscess/fistula drainage may improve wound healing with a low rate of fecal diversion.^11^ Whether or not azathioprine and anti-TNF-α agents increase the risk of surgical complications is debated.^32–34^ One previous study implemented similar preoperative management, the rates of complications is similar with ours.^11^ Our results of CD recurrence are comparable with those reported in previous studies.^35^ These should be associated with smoking cessation, postoperative enteral nutrition, and azathioprine maintenance therapy. As smoking were identified as predictors of recurrent disease activity, and enteral nutrition and azathioprine are both effective treatment to maintain CD remission.^14,35^ The preoperative nutritional therapy may have no influence on the rate of recurrence events.

The rate of temporary fecal diversion in our study is 22.0% (20/91), which is higher than one previous study.^11^ Rates of temporary diverting stoma in CD reported are various, from 7.7% to 51%.^4,11^ The difference in stoma rates may result from kinds of indications for stoma adopted by different surgeon teams. Zerbib et al^11^ performed temporary diverting stoma “if an abscess was present at the time of surgery or in case of complex ileosigmoid fistula requiring a large sigmoidectomy.” While our indication was that presence of abscess irrespective of drainage before surgery, and/or severe intestinal edema, and/or ≥2 anastomosis performed.

There are several limitations of our study. First, as the definition of improvement of malnutrition is not unanimous, we defined it as serum albumin >35 g/L and gain of body weight >3 kg based on our experience. Whether or not different definitions affect the outcome is not clear. Second, we did not enroll well-nourished patients and patients with normal serum CRP, so whether these patients could benefit from preoperative nutrition and what end-points are suitable for them remain unclear. Finally, not all patients respond to nutritional therapy in terms of inflammation regulation, and some patients will need a very long duration of nutrition treatment to get benefit. The end-points used in this study may not be suitable for these patients.

In conclusion, preoperative nutritional therapy for CD patients who underwent resection could reduce the rate of postoperative morbidity compared with the previous studies. ROI (normalization of serum CRP) as the end-point of preoperative nutritional therapy could be achieved in a much shorter time compared with IOM, and have the same effects in terms of reducing postoperative complications, temporary fecal diversion, and disease recurrence. Therefore, ROI is a better end-point of preoperative nutritional therapy than IOM in CD patients requiring bowel resection.
